# At the host-pathogen interface: tomato phosphoinositide-specific phospholipase C2 promotes *Phytophthora infestans* colonization

**DOI:** 10.1093/plphys/kiag614

**Published:** 2026-08-18

**Authors:** Mamoona Khan

**Affiliations:** Assistant Features Editor, Plant Physiology, American Society of Plant Biologists; Department of Plant Pathology, Institute of Crop Science and Resource Conservation (INRES), University of Bonn, Nussallee 9, 53115 Bonn, Germany

Phosphoinositides (PIs) are low-abundance but highly dynamic membrane components that regulate membrane identity, trafficking and cellular signaling (Posor et al. 2022). Their abundance and subcellular distributions change in response to environmental cues, thereby enabling membranes to recruit specific proteins and coordinate cellular responses, including plant immunity (Heilmann and Heilmann 2022; Zarreen et al. 2023).

Phosphoinositide-specific phospholipase C (PI-PLC) hydrolyzes phosphatidylinositol 4-phosphate (PI4P) and phosphatidylinositol 4,5-bisphosphate (PI(4,5)P_2_), initiating cellular signaling. Multiple studies have implicated plant PI-PLCs in plant–pathogenic fungal interactions, where they can contribute to immune responses and disease resistance (Fang et al. 2023). However, their activity does not invariably favor the plant. In tomato (*Solanum lycopersicum*), loss of *PLC2* (*SlPLC2*) reduced susceptibility to the necrotrophic fungus *Botrytis cinerea*, thereby identifying it as a host susceptibility factor (Perk et al. 2023). Whether SlPLC2 similarly promotes infection by other pathogens, however, remained unclear.

The hemibiotrophic oomycete *Phytophthora infestans*, the causal agent of late blight, establishes specialized host-derived membrane interfaces during initial colonization of living host cells (during the biotrophic phase; Fig. 1). Following penetration, an intracellular infection vesicle forms from which invasive hyphae emerge, while subsequently formed haustoria are surrounded by the extrahaustorial membrane (EHM). These interfaces undergo extensive protein and lipid reorganization (Bozkurt et al. 2014); however, the host lipid-modifying enzymes involved remain largely unknown.

**Figure 1:**
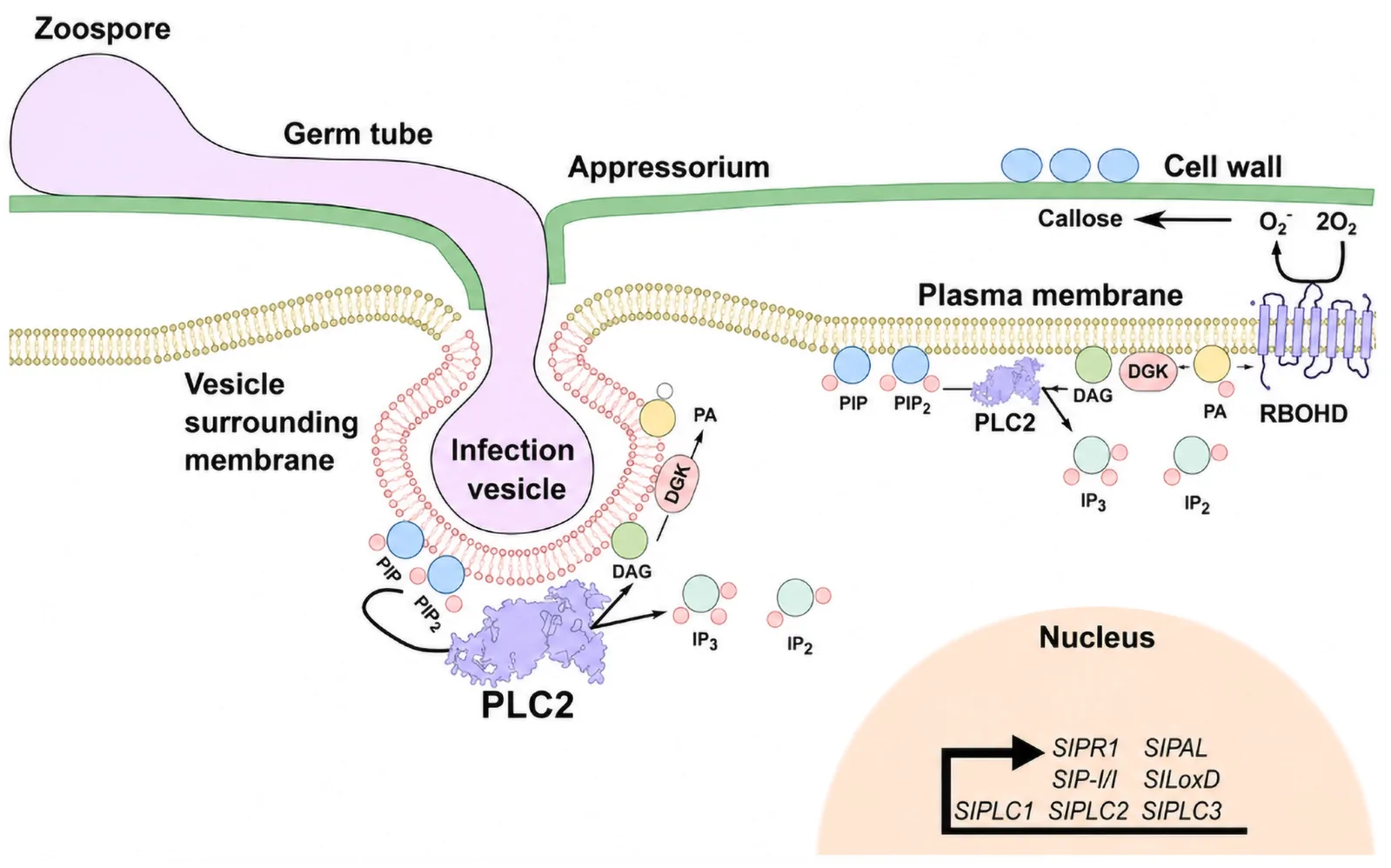
Proposed model of SlPLC2 function during *Phytophthora infestans* infection, adapted from Perk et al (2026). Following host penetration, *P. infestans* forms an intracellular infection vesicle associated with early defense responses, including ROS production and callose deposition. SlPLC2 localizes to the plasma membrane and membranes surrounding infection vesicles, where its potential substrates PI4P and PI(4,5)P_2_ are also present. Local phosphoinositide turnover may influence membrane-associated signaling and facilitate early pathogen establishment.

In a (Perk et al. 2026, publication date 18.08) issue of *Plant Physiology*, Perk et al. (2026) investigated the role of SlPLC2 during *P. infestans* infection. Their key finding is that *SlPLC2* acts as a host susceptibility factor that promotes early pathogen colonization and localizes to membrane interfaces associated with the initial stages of infection. The study therefore extends the susceptibility function of SlPLC2 from the necrotrophic fungus *B. cinerea* to the hemibiotrophic oomycete *P. infestans*.

The authors first examined whether *SlPLC2* responds transcriptionally to *P. infestans* infection. Among the 6 tomato PI-PLC genes, *SlPLC2* was significantly induced around infection sites during the early stages of infection. This local induction raised the possibility that *SlPLC2* contributes to *P. infestans* colonization. To test this hypothesis, the authors analyzed 2 independent CRISPR-Cas9 *SlPLC2* knockout lines. Both lines displayed reduced susceptibility to the pathogen, with smaller lesions, lower pathogen biomass, and significantly fewer sporangia than wild-type plants. This phenotype was reproduced with 2 genetically distinct *P. infestans* isolates, indicating that the contribution of *SlPLC2* to susceptibility is not isolate-specific.

To determine whether the reduced susceptibility of *SlPLC2* knockout plants was associated with altered defense signaling, Perk et al. (2026) examined salicylic acid (SA)- and jasmonic acid (JA)-associated responses during infection. Compared with wild-type plants, the knockout lines showed reduced or temporally altered expression of several SA- and JA-associated marker genes and lower JA accumulation, whereas SA levels remained comparable between genotypes. Thus, reduced susceptibility was not associated with stronger SA- or JA-related defense responses. The authors also examined ROS production and callose deposition, 2 additional defense-associated responses. Both responses were strongly reduced in *SlPLC2* knockout plants. These findings presented an intriguing contrast to previous results, that is, loss of *SlPLC2* restricted pathogen growth despite attenuating several defense-associated responses.

This apparent paradox raised the possibility that the attenuated defense responses were a consequence of impaired pathogen establishment rather than the cause of reduced susceptibility. To address this possibility, the authors examined the early stages of *P. infestans* infection by microscopy. Confocal imaging revealed significantly fewer infection vesicles in the knockout lines than in wild-type plants, indicating impaired early pathogen establishment. Consistent with this result, induction of the pathogen-produced biotrophy-associated effector *PiAvrblb2* was delayed in the knockout plants, and pathogen transcript accumulation remained lower at later stages of infection. These findings suggest that *P. infestans* colonization is delayed or impaired in the absence of *SlPLC2* and that the attenuated defense-associated responses may, at least in part, reflect reduced pathogen establishment.

A key insight into how SlPLC2 might promote early colonization came from examining its subcellular localization during infection. Whereas SlPLC2-GFP was predominantly associated with the plasma membrane in uninfected cells, during infection it was also detected at host-derived membranes surrounding infection vesicles. Biosensors for the PI-PLC substrates PI4P and PI(4,5)P_2_ also labeled these membranes, with SlPLC2 showing spatial overlap with PI4P-positive structures. Thus, SlPLC2 is positioned at an early infection interface where its potential phosphoinositide substrates are present.

Altogether, the study by Perk et al. (2026) extends the susceptibility function of SlPLC2 from *B. cinerea* to *P. infestans* and points to early infection-associated membranes as a potential site of its action. The presence of SlPLC2 and its potential substrates PI4P and PI(4,5)P_2_ at these membranes raises the possibility that SlPLC2 modifies the local lipid environment during pathogen entry. By hydrolyzing these phosphoinositides, SlPLC2 could alter membrane lipid composition and downstream signaling in ways that favor pathogen establishment. Whether SlPLC2 is enzymatically active at these interfaces, however, remains unknown. Monitoring PI4P and PI(4,5)P_2_ dynamics in wild-type and SlPLC2 knockout plants during pathogen entry could help test this model. Similarly, understanding how SlPLC2 facilitates early colonization may ultimately reveal how pathogens with contrasting lifestyles exploit a common host lipid-signaling component.

## Recent related articles in *Plant Physiology*

- D'Ambrosio et al., (2017) identified *Arabidopsis thaliana* PLC2 as a component of MAMP-triggered immunity and nonhost resistance.
- Laxalt et al. (2025) reviewed current knowledge of plant PI-PLC signaling and highlighted its plant-specific features, downstream second messengers, and roles in development and stress responses.
- Heilmann and Heilmann (2025) reviewed how PtdIns(4,5)P_2_ functions as a regulatory membrane lipid in plants, with particular emphasis on its dynamic distribution and integration into plant signaling networks.

## Data Availability

No data were generated or analyzed in this study.
